# Further characterization of the *BRSK2*-associated neurodevelopmental disorder

**DOI:** 10.1038/s41431-026-02195-7

**Published:** 2026-07-27

**Authors:** Palak Singhal, Tzung-Chien Hsieh, Nadja Ehmke, Elena Bacchelli, Marta Viggiano, Elena Maestrini, Paola Visconti, Annio Posar, Maria Cristina Scaduto, Alessandro Vaisfeld, Carey Ronspies, Sarah Burke, Joana Rosmaninho Salgado, Joaquim Sá, Sara Ribeiro, Amelle Shillington, Anjali Aggarwal, Christina Dailey, Carol Saunders, Florencia Del Viso, Chaya N. Murali, Melissa MacPherson, Oana Caluseriu, Alain Verloes, Jonathan Levy, Yline Capri, Hannah S. Kemmer, Manuel Holtgrewe, Philip M. Boone, Lance Rodan, Georgia Vasileiou, Melissa Pauly, André Reis, Isabella Herman, Ivy Johnson, Himanshu Goel, Ana Maria Rodriguez Barreto, Flavio Faletra, Catia Mio, Mona L. Essawi, Heba A. Hassan, Wessam E. Sharaf-Eldin, Nirmeen Kishk, Giuseppe Donato Mangano, Renata Mangano, Andrea K. Shields, Judith D. Ranells, Trine Bjørg Hammer, Clara Velmans, Christian Netzer, Nora Winnerling, Konstantinos Kolokotronis, Benjamin Seidl, Anita Rauch, Alberto Fernandez-Jaen, Aboulfazl Rad, Gabriela Oprea, Paskal Cullufi, Sonila Tomori, Claire Beneteau, Marine Legendre, Caroline Rooryck, Hannah Klinkhammer, Tobias B. Haack, Amjad Khan, Johanna Kick, Deborah Bartholdi, Dominique Braun, Erin E. Baldwin, David H. Viskochil, Lorenzo D. Botto, Anna LaGroon, Emily Black, Kameryn M. Butler, Emmanuelle Ranza, Manon Macherel, Vincent Desportes, Mathilde Pujalte, Louis Januel, Boris Keren, Cyril Mignot, Madeleine Harion, Maartje L. E. Voors, Charlotte W. Ockeloen, Javier Porta-Pelayo, Bernt Popp, Peter Krawitz, Heinrich Sticht, Anne Gregor, Christiane Zweier

**Affiliations:** 1 Department of Human Genetics, Inselspital Bern, University of Bern, Bern, Switzerland; 2 Department for Biomedical Research (DBMR), University of Bern, Bern, Switzerland; 3 Graduate School for Cellular and Biomedical Sciences, University of Bern, Bern, Switzerland; 4 Medical Faculty, Institute for Genomic Statistics and Bioinformatics, University of Bonn, Bonn, Germany; 5 Institute of Medical Genetics and Human Genetics, Charité - Universitätsmedizin Berlin, Berlin, Germany; 6 Department of Pharmacy and Biotechnology, University of Bologna, Bologna, Italy; 7 IRCCS Istituto delle Scienze Neurologiche di Bologna, Bologna, Italy; 8 IRCCS Istituto delle Scienze Neurologiche di Bologna, UOSI Disturbi dello Spettro Autistico, Bologna, Italy; 9 Department of Biomedical and Neuromotor Sciences, University of Bologna, Bologna, Italy; 10 Department of Medical and Surgical Sciences, University of Bologna, Bologna, Italy; 11 Medical Genetics Unit, IRCCS Azienda Ospedaliero-Universitaria di Bologna, Bologna, Italy; 12 Munroe-Meyer Institute, University of Nebraska Medical Center, Omaha, Nebraska USA; 13 Medical Genetics Unit, Hospital Pediátrico, Centro Hospitalar e Universitário de Coimbra, Coimbra, Portugal; 14 Cincinnati Children’s Hospital Medical Center, Department of Human Genetics, Cincinnati, OH USA; 15 Division of Genetics and Metabolism, Department of Pediatrics, University of Minnesota, Minneapolis, MN USA; 16 Department of Pathology and Laboratory Medicine, Children’s Mercy Hospital, Kansas City, MO USA; 17 Genomic Medicine Center, Children’s Mercy Hospital, Kansas City, MO USA; 18 Department of Pediatrics and Pathology, School of Medicine, University of Missouri, Kansas City, MO USA; 19 Department of Molecular and Human Genetics, Baylor College of Medicine, Houston, TX USA; 20 Department of Medical Genetics, Faculty of Medicine and Dentistry, University of Alberta, Alberta Health Services, Edmonton Zone, Edmonton, AB Canada; 21 Genetics Department, Robert Debré-APHP Nord-Université Paris Cité, ERN-ITHACA, Paris, France; 22 Freie Universität Berlin, Berlin, Germany; 23 Humboldt-Universität zu Berlin, Berlin, Germany; 24 Berlin Institute of Health, Charité - Universitätsmedizin Berlin, Berlin, Germany; 25 Division of Genetics and Genomics, Boston Children’s Hospital, Harvard Medical School, Boston, MA USA; 26 Program in Medical and Population Genetics, Broad Institute of MIT and Harvard, Cambridge, MA USA; 27 Center for Genomic Medicine, Massachusetts General Hospital, Boston, MA USA; 28 Department of Neurology, Boston Children’s Hospital, Boston, MA USA; 29 Institute of Human Genetics, Universitätsklinikum Erlangen, Friedrich-Alexander-Universität Erlangen-Nürnberg, Erlangen, Germany; 30 Centre for Rare Diseases Erlangen (ZSEER), Universitätsklinikum Erlangen, Erlangen, Germany; 31 Boys Town National Research Hospital, Boys Town, NE USA; 32 Munroe-Meyer Institute for Genetics and Rehabilitation, University of Nebraska Medical Center, Omaha, NE USA; 33 Hunter Genetics, Newcastle, NSW Australia; 34 Division of Clinical Genetics and Metabolism, Nicklaus Children’s Hospital, Miami, FL USA; 35 Institute of Medical Genetics, University Hospital ‘Santa Maria della Misericordia’ (ASUFC), Udine, Italy; 36 Department of Medicine, University of Udine, Udine, Italy; 37 Medical Molecular Genetics Department, Human Genetics and Genome Research Institute, National Research Centre, Cairo, Egypt; 38 Neurology Department, Faculty of Medicine, Cairo University, Cairo, Egypt; 39 Department of Medicine and Surgery, University of Enna “Kore”, Enna, Italy; 40 Neuropsychology Lab, Department of Psychology, Educational Science and Human Movement, University of Palermo, Palermo, PA Italy; 41 Department of Pediatrics, Division of Genetics and Metabolism, University of South Florida, Tampa, FL USA; 42 Department of Clinical Genetics, Copenhagen University Hospital, Copenhagen, Denmark; 43 Department of Epilepsy Genetics and Personalized Treatment, The Danish Epilepsy Centre, Dianalund, Denmark; 44 Institute of Human Genetics, Faculty of Medicine and University Hospital of Cologne, Cologne, Germany; 45 Institute of Medical Genetics, University of Zurich, Schlieren, Zurich, Switzerland; 46 Research Priority Program (URPP) ITINERARE: Innovative Therapies in Rare Diseases, University of Zurich, Zurich, Switzerland; 47 Neuroscience Center Zurich, University of Zurich and ETH Zurich, Zurich, Switzerland; 48 Servicio de Neurología Infantil, Sección de Neurogenética, Hospital Universitario Quirónsalud, Madrid, España; 49 Facultad de Medicina, Universidad Europea de Madrid, Madrid, España; 50 Arcensus GmbH, Rostock, Germany; 51 Pediatric Department, University Hospital Center “Mother Teresa”, Tirana, Albania; 52 CHU Bordeaux, Service de Génétique Médicale, F-33000 Bordeaux, France; 53 Institute for Medical Biometry and Statistics, Marburg University, Marburg, Germany; 54 Institute of Medical Genetics and Applied Genomics, University of Tübingen, Tübingen, Germany; 55 Department of Zoology, University of Lakki Marwat, Khyber Pakhtunkhwa, Pakistan; 56 Division of Medical Genetics, Department of Pediatrics, University of Utah, Salt Lake City, UT USA; 57 Greenwood Genetic Center, Greenwood, SC USA; 58 Medigenome, Swiss Institute of Genomic Medicine, Geneva, Switzerland; 59 Developmental pediatrics practice, Lausanne, Switzerland; 60 Centre de Référence des Maladies Rares et Déficience Intellectuelle, HCL Université de Lyon, Service de Neuropédiatrie HFME, Lyon, France; 61 Department of Genetics, Lyon University Hospital, Lyon, France; 62 APHP Sorbonne Université, Département de Génétique, Groupe Hospitalier Pitié-Salpêtrière and Hôpital Armand Trousseau and Centre de Référence Déficiences Intellectuelles de Causes Rares Paris, Paris, France; 63 APHP Sorbonne Université, Service de Neuropédiatrie, Hôpital Armand Trousseau, Paris, France; 64 Department of Human Genetics, Radboud University Medical Center, Nijmegen, The Netherlands; 65 Genologica Center, Málaga, Spain; 66 Center of Functional Genomics, Berlin Institute of Health at Charité, Universitätsmedizin Berlin, Berlin, Germany; 67 Institute of Human Genetics, University of Leipzig Medical Center, Leipzig, Germany; 68 Institut für Biochemie, Friedrich-Alexander-Universität Erlangen-Nürnberg, Erlangen, Germany

**Keywords:** Genetics research, Clinical genetics

## Abstract

Variants in *BRSK2*, encoding brain specific kinase-2, have recently been associated with an autosomal dominant neurodevelopmental disorder (NDD). We have assembled 52 cases with heterozygous *BRSK2* variants and variable neurodevelopmental phenotypes with frequent neuropsychiatric and behavioral symptoms. The variant spectrum included 15 different truncating variants, seven (potential) splice variants, three structural variants, and 12 different missense variants. Of the missense variants, seven were in the kinase domain, and the others in the UBA and the KA1 domain or outside domains. Variants occurred de novo in 19 cases and were inherited in 18. We utilized *Drosophila melanogaster* as a model and assessed viability and performed climbing and bang sensitivity assays upon knockdown of the fly orthologue *sff* or upon overexpression of wildtype or mutant human *BRSK2*. Pan-neuronal knockdown of *sff* resulted in impaired locomotor behavior and seizure susceptibility. Ubiquitous or pan-neuronal overexpression of human wildtype *BRSK2* in *Drosophila* resulted in lethality or locomotor impairment, respectively, indicating toxicity. Overexpressing mutant *BRSK2* did not or incompletely affect viability and locomotor behavior for six of seven tested kinase domain missense variants and one KA1 domain variant, indicating a (partial) loss-of-function effect. Interestingly, overexpressing BRSK2 with the remaining missense variant from the kinase domain and the two most C-terminal missense variants resulted in possible gain of function. Our findings further delineate the clinical and molecular spectrum of *BRSK2*-associated NDD and provide further insights into the role of BRSK2/sff in nervous system function and dysfunction.

## Introduction

BRSK2 (MIM: 609236) is located on chromosome 11p15.5 and is highly expressed in the human brain [1]. It encodes brain specific kinase-2, a mammalian serine/threonine kinase that belongs to the AMP-activated protein kinase (AMPK)-related subfamily of protein kinases [2]. It is evolutionarily conserved with existing orthologs in mice (Brsk2), *Drosophila* (*sff*), *C. elegans* (sad-1) and Zebrafish (brsk2b). BRSK2 contains a highly conserved N-terminal protein kinase domain, a Ubiquitin-associated (UBA) domain, and a C-terminal KA1 (Kinase-associated1) domain. The latter includes a KEN box, a motif sequence targeted by anaphase promoting complex (APC), a ubiquitin protein ligase with consensus sequence K-E-N-x-x-x-N [3]. Together with its brain specific kinase paralog, BRSK1 (MIM: 609235), BRSK2 is involved in two major neuronal processes, axonogenesis and polarization [4]. Upon phosphorylation by LKB1 (liver kinase B1), BRSK2 controls microtubule dynamics by phosphorylating tau, thus contributing to axon specification and arborization [5]. Another substrate of BRSK2 is PAK1 (MIM: 602590) [6], a kinase which is also implicated in a neurodevelopmental disorder (IDDMSSD; MIM#618158). Furthermore, BRSK2 phosphorylates TSC2 (MIM: 191092) and PTEN (MIM: 601728) and is thus involved in the regulation of the mTOR signaling pathway [7], a pivotal cascade in cell growth, metabolism and neural development.

Clinical relevance of *BRSK2* has recently emerged through several studies, which have reported de novo truncating or missense variants in 39 individuals with variable neurodevelopmental phenotypes, including intellectual disability (ID) and autism spectrum disorder (ASD) [8–21], often without providing detailed clinical information. So far, no specific OMIM disease entry has been assigned to BRSK2.

In this study, we report 52 individuals with *BRSK2* variants, thus further delineating the molecular and clinical spectrum of *BRSK2*-associated NDD. In *Drosophila*, we demonstrate a crucial role of its ortholog, *sugar free frosting* (*sff*), in development and neurological function and show evidence for a (partial) loss-of-function effect for six of seven missense variants from the kinase domain and for one variant from the KA1 domain.

## Material and methods

### Data collection

Through international collaboration via Genematcher, VarSome, DECIPHER, ERN ITHACA and scientific communication, 52 cases with variants in *BRSK2* were assembled, and standardized clinical and molecular details of the affected individuals were collected via a questionnaire. Testing in collaborating centers was conducted either as part of standard diagnostic procedures, exempt from institutional ethics approval, or within research studies approved by the ethical review boards of the respective institutions (e.g., a study for rare diseases is approved by the cantonal ethical board Bern (KEK), project ID 2021-01396) (Supplementary Table S1). The study complied with the principles set out in the Declaration of Helsinki. Consent for publication of molecular and clinical data (with or without photographs) and/or submission to GestaltMatcher was obtained from either the probands, their parents, or legal guardians.

Identified variants are annotated based on the MANE select transcript of *BRSK2* (GenBank: NM_001256627.2, NP_001243556.1) and were classified according to ACMG criteria and current recommendations from the Clinical Genome Resource (ClinGen; www.clinicalgenome.org). Variants were submitted to ClinVar (accession numbers SCV007592125-SCV007592158).

### In silico analyses

Splice-site or missense variants close to a splice site were tested for their potential effect on splicing using SpliceAI and Pangolin (Supplementary Table S2). Franklin (https://franklin.genoox.com) was utilized to predict pathogenic effects of the missense variants with different tools (Supplementary Table S2). Alignments for conservation of amino acids affected by missense variants were performed using Clustalw2.

### Structural modeling

Structural analysis of BRSK2 and molecular modeling of sequence variants was based on the crystal structure of murine BRSK2 (PDB: 4yom) [22] that exhibits 99% sequence identity to human BRSK2. Functionally important sequence stretches were identified from searches in the Prosite and ELM database. Variant modeling was done with SwissModel [23], and RasMol [24] was used for structure analysis and visualization. Potential pathogenicity of variants was quantified using AlphaMissense.

### Facial gestalt and similarity analysis

We evaluated facial similarity using the GestaltMatcher framework [25, 26] as described in more detail in the Supplementary Methods.

### Fly lines and conditions

*Sugar free frosting* (*sff*) is the well conserved *Drosophila* orthologue of *BRSK2* and *BRSK1*, with DIOPT (https://www.flyrnai.org/cgi-bin/DRSC_orthologs.pl) scores of 12, and 49% and 53% sequence identity, respectively. Conservation is even higher within domains, particularly for the kinase domain with 89% (Supplementary Fig. S1).

Flies were raised on standard food containing cornmeal, sugar, agar and yeast. Tissue- specific knockdown or overexpression was achieved with the UAS/GAL4 system [27]. Driver lines were obtained from the Bloomington Drosophila Stock Center (Indiana University, Bloomington, USA) or assembled in house and by colleagues (BL#7415; *repo*-GAL4/Tm3Sb [glial expression], BL#8765, *elav*-GAL4/Cyo [pan-neuronal expression], *actin*-GAL4/Tm3Sb Tb [ubiquitous expression]). To take into account potential variability in knockdown efficiency or off-target effects, three independent RNAi lines targeting *sff* (RNAi lines 1–3) were used in this study, each carrying a distinct double-stranded RNA transgene inserted at a different genomic location. RNAi lines were obtained from the Vienna Drosophila Research Center (VDRC, Vienna, Austria) (RNAi-line 1:100717/KK and 60100 [Control 1];) and from Bloomington Stock Center (RNAi-line 2: BL#36656, RNAi-line 3: BL#55897and BL#36303 [Control 2]) (Supplementary Table S3). For the generation of UAS-driven overexpression lines, *BRSK2* was cloned from a Sino Biological (Bejing, China) vector No. #HG20334-UT into a TOPO2.1 vector (Thermo Fisher Scientific, Waltham, MA, USA). Site-directed mutagenesis was performed as described previously [28] for eleven missense variants from our cohort. Constructs were then subcloned into a pUAST-attb fly expression vector (Stock 1419; DGRC, Indiana University, Bloomington, IN, USA). Primer sequences are provided in Supplementary Table S4. After sequence verification of the clones, constructs were sent to FlyORF (Zurich, Switzerland) for injection into the line BL#24749 (Fly Control) to create transgenic flies. Fly lines overexpressing BRSK2 containing missense variant p.(Gly28Arg) or p.(Ala158Thr) from the kinase domain or p.(Lys603Glu) from the C-terminal KA1 domain correspond to OE_WTBRSK2_1 from the same injection batch, and fly lines overexpressing BRSK2 containing one of the other eight missense variants correspond to OE_WTBRSK2_2, generated under identical conditions (Supplementary Table S3). Double transgenic fly lines (*sff* RNAi-line 2 combined with OE_WTBRSK2 or OE_mutantBRSK2) were generated by multiple crossing steps using a double balancer line (Kr/CyO;D/Tm6c Sb Tb) (Supplementary Table S3).

Ubiquitous (*actin*-GAL4) knockdown (*sff* RNAi-lines 1-3) or overexpression (OE_WTBRSK2 and OE_mutantBRSK2) was confirmed with quantitative real-time PCR as described elsewhere [28] after RNA isolation from larvae (4-5 L3 larvae per genotype were used) (Supplementary Fig. S2).

### Viability analysis

Viability was assessed by counting adult flies every 24 h until no more offspring hatched. Pupae were counted after no more offspring hatched. To test for pupal development at different stages, light, dark and empty non-Tb pupae were counted. Statistical testing was done with chi-square test. A (partial) lethality phenotype was determined when numbers of *sff* deficient or *BRSK2* overexpressing flies was significantly different from the expected ratio considering the segregation of balancer chromosomes and the respective corresponding control lines.

### Behavioral experiments

*Sff* RNAi lines or *BRSK2* overexpression lines or *BRSK2* OE/*sff* RNAi double balancer lines and their corresponding genetic background control lines were crossed to the *elav*-GAL4/Cyo (pan-neuronal) or to the *repo*-GAL4/Tm3Sb (glial) driver lines. Seizure susceptibility in the offspring was tested with the bang sensitivity assay as described in more detail in the Supplementary methods.

## Results

### Molecular spectrum of variants in *BRSK2*

**Fig. 1:**
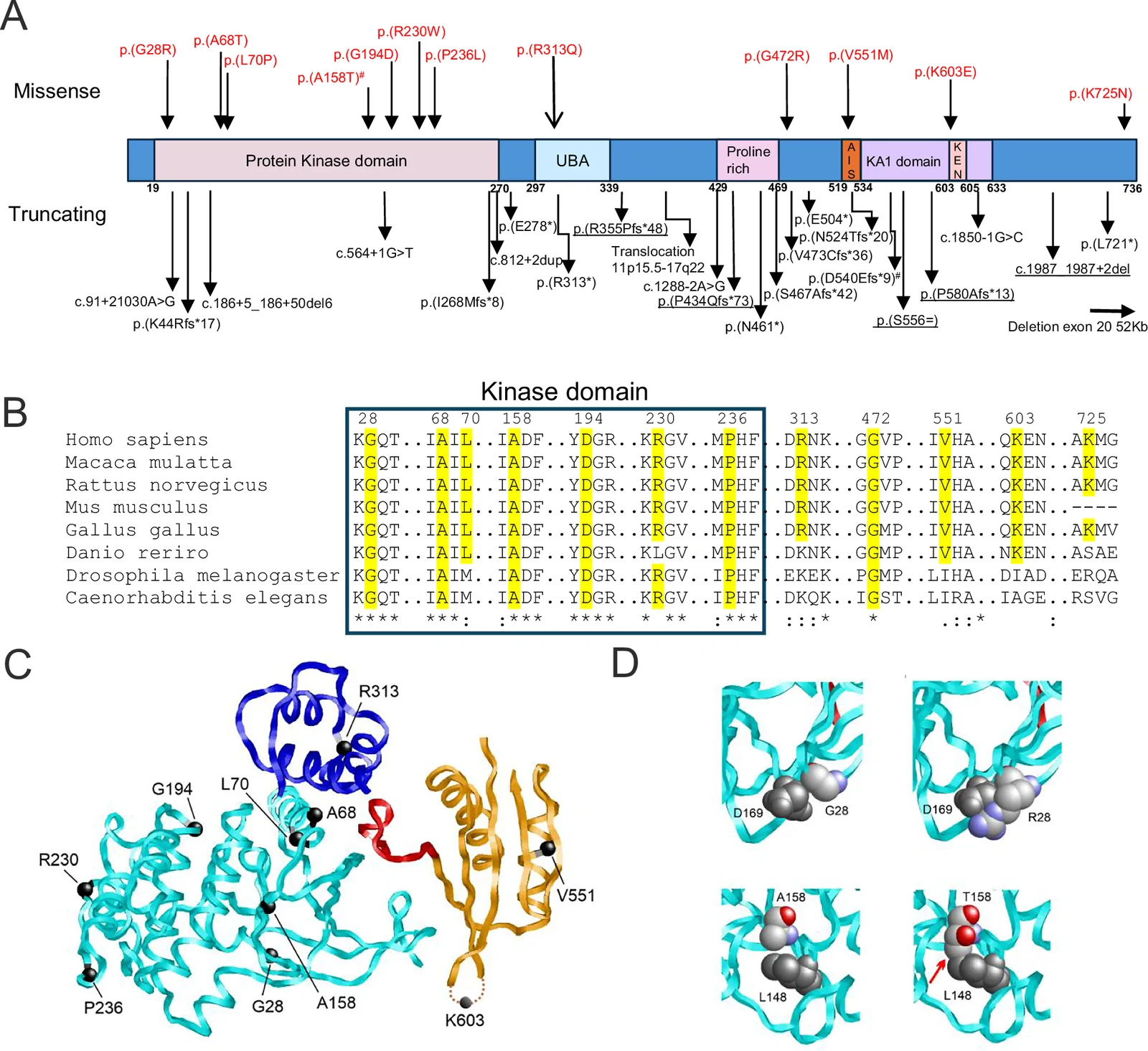
Overview of published and novel variants in *BRSK2*. **A** Schematic drawing of BRSK2 (NM_001256627.2, NP_001243556.1) with domains according to Uniprot/Interpro and with annotation of variants from this study. Variants of two previously published cases [19] are indicated by #. Truncating variants below the scheme are indicated in black, and missense variants above the scheme in red. The underlined variants are recurrent variants observed in two or more independent individuals/families in this cohort. Two of them (p.(Pro434GlnfsTer73) and p.(Pro580ArgfsTer13) have additionally been reported in independent cases in previous studies [13, 21]. **B** Conservation of BRSK2 amino acid residues affected by missense variants. All affected residues are mostly conserved in vertebrates. Variant positions are highlighted in yellow. Alignment was performed with Clustalw2 with the following reference sequences: *Homo sapiens*: NP_001243556.1, *Macaca mulatta*: AFI38697.1, *Rattus norvegicus*: NP_001406495.1, *Mus musculus*: NP_001404016.1, *Gallus gallus*: NP_001186525.2, *Danio rerio*: XP_017209740.1, *Drosophila melanogaster*: NP_648814.3, *Caenoherbiditis elegans:* NP_001076761.1. Variants within the box are from the kinase domain. **C** Crystal structure of BRSK2 showing the postion of the residues for which sequence variants were found in the present study. The structure is colored according to the domain architecture (kinase domain, cyan; UBA domain, blue; AIS region, red; KA1 domain, orange). G472, K603 and K725 are located in flexible regions that are not resolved in the structure. The loop harboring K603 is schmetically indicated as a dotted line. **D** (Top row) Wildtype G28 forms weak interactions with D169. In the G28R variant, R28 tightly interacts with the oppositely charged D169 thereby affecting the structure of the ATP-binding site (residues 25–33). (Bottom row) Wildtype A158 forms weak interactions with L148. Regarding the A158T variant, the bulkier T158 sidechain causes steric clashes with L148 (red arrow), which are predicted to decrease stability of the kinase domain.

This cohort comprises 52 cases from 44 families, of which 50 are novel, and two were previously published [19]. In 19 cases, variants occurred de novo, in 18 the variant was inherited, and in 15 parents were not available for testing. In individual 12, the variant was also found in 4 of 215 sequencing reads in paternal blood. Though it was not found in 222 reads from buccal swab DNA, paternal low-grade mosaicism is suspected. Eight of the inherited variants were transmitted from a parent with a reported neurodevelopmental phenotype, while in the other cases, the transmitting parent was either apparently unaffected or parental phenotypic information was unavailable. A total of 37 different *BRSK2* variants (Supplementary Table S1, Fig. 1A) comprised three structural variants (SV) as well as 12 missense, 15 truncating and seven potential splicing variants. Of the SVs, one deletion affected the C-terminal exon(s) of *BRSK2* and adjacent *MOB2* (MIM: 611969), while the other additionally affected *MOB2*, *DUSP8* (MIM: 602038), and part of *KRTAP5-1* (MIM: 148022). The translocation disrupts *BRSK2* (GRCh38:chr11:1446815-1446816) with a 4 bp deletion at the breakpoint in intron 12. Truncating variants included frameshift and nonsense variants and were distributed across the gene. Three of them (p.(Arg355ProfsTer48), p.(Pro434GlnfsTer73), p.(Pro580AlafsTer13)) were identified recurrently in two or three independent individuals/families, respectively. Variants p.(Pro434GlnfsTer73) and p.(Pro580ArgfsTer13) also have been reported in other individuals with NDD or ASD previously [13, 21]. Six of seven potential splice variants were predicted to have a strong effect on splicing by SpliceAI and/or Pangolin (Supplementary Table S2). Variants in canonical splice sites are predicted to lead to either out-of-frame (c.564+1 G > T, c.812+2dup and c.1288-2 A > G) or in-frame (c.1850-1 G > C, and p.(Ser556 = )) exon skipping. Impact remains unclear for two deep intronic variants (c.91+21030 A > G, c.186+5_186+50del). Notably, variant c.91+21030 A > G affects the start codon of an alternative transcript (NM_001256630.1) (Supplementary Table S1). Two (potentially) truncating variants, c.1987_1987+2del and p.(Lys721*), were in the splice site of the penultimate and in the last exon, respectively, thus likely escaping nonsense mediated mRNA decay (Fig. 1A).

Seven of the twelve identified missense variants reside in the kinase domain, and five in other or outside domains (Fig. 1A). Missense variants from the kinase domain were predicted to be deleterious by several in silico prediction tools (Supplementary Table S2) and affected highly conserved amino acid residues (Fig. 1B). In contrast, missense variants from the UBA and KA1 domains or outside domains affected amino acid residues which were conserved only across most vertebrates (Fig. 1B), and in silico tools predicted at least two of them as not deleterious (Supplementary Table S2). While the mean CADD score was identical for kinase domain variants (mean CADD 26.1, range 23.6–28.5) compared to non-kinase variants (mean CADD 26.1, range 21.5–29.4), AlphaMissense scores were consistently higher for kinase domain variants (mean 0.956, range 0.644–0.9999) compared to non-kinase domain variants (mean 0.614, range 0.174–0.947). Similarly, PrimateAI-3D scores, all assessed against a gene-specific threshold of 0.55, were higher on average for kinase domain variants (mean 0.836, range 0.70–0.99) than for non-kinase domain variants (mean 0.752, range 0.65–0.86) (Supplementary Table S2).

Twenty-two variants were absent from gnomAD [29], while twelve variants were reported with low allele frequencies ( < 0.000001, allele counts between 1x and 26x) (Supplementary Table S1). According to ACMG criteria and current ClinGen recommendations, 17 variants were classified as likely pathogenic or pathogenic, and 17 variants as variants of unknown significance (VUS) (Supplementary Table S1). *BRSK2* is predicted to be highly intolerant towards loss-of-function variants (pLI=1, observed/ expected = 0.24, LoeUF= 0.36), while less intolerant for missense variants (z = 2.13, o/e = 0.81) [29]. Additional variants in other disease-associated genes were observed in 15 individuals, several of them in genes associated with an NDD (Supplementary Table S5).

### Structural modeling of *BRSK2* missense variants

Structural analysis with AlphaMissense [30] predicted all variants in the kinase domain and variant p.(Val551Met) in the KA1 domain as pathogenic due to significant impairment of the domain structure and subsequent loss of function (Supplementary Table S2). Location of variants within the crystal structure of BRSK2 are shown in Fig. 1C, and structural effects of the p.(Gly28Arg) and p.(Ala158Thr) variants from the kinase domain are exemplarily shown in Fig. 1D. Arg313 is located in the UBA domain which has auto-regulatory function for the kinase domain [22]. The Arg313Gln exchange disrupts a salt bridge that is formed in the wildtype between Arg313 and Glu57 of the kinase domain and might therefore affect the regulatory function of the UBA domain. The variants Gly472 Arg, Lys603Glu and Lys725Asn are located outside the globular domains and are therefore not expected to have a negative effect on BRSK2 structure itself, which is in line with the low AlphaMissense scores for the latter two. However, such nonglobular parts of proteins frequently contain so-called ‘linear sequence motifs’ that play an important role for protein-protein interactions [31]. Gly472 resides within a region (Ser423-Ser513) with a high phosphorylation rate of critical serine/threonine residues and close to a D-box-motif (RxxLxxxxN, Arg476-Asn484), a mark for ubiquitination by the anaphase-promoting-complex/cyclosome (APC/C) E3-ubiquitin-ligase [32]. Lys603 is part of a lysine-glutamate-asparagine (K-E-N) sequence motif. This motif of BRSK2 was also shown to be required for APC/C-Cdh1 dependent degradation [33]. Lys725 is a conserved residue of a putative USP7 binding motif suggesting that BRSK2 might exert further functions in ubiquitin-proteasome pathway. All three variants destroy or might impair the respective sequence motifs and could therefore affect the function of BRSK2 in protein-protein interactions.

### Clinical phenotypes of individuals with variants in *BRSK2*

All but nine of the affected individuals in this study were reported with variable degrees of ID and/or developmental delay. Most individuals for whom detailed clinical information was available had speech delay (37/45), while motor delay was reported in 17/43 individuals. If information on ID was available, severity ranged from borderline to moderate. Nine individuals had no apparent ID and/or developmental delay. Four were parents or siblings of an index case, and the other five were tested due to behavioral abnormalities or were too young to assess cognitive abilities. Behavioral issues were reported in 43/50 individuals and included ASD, attention deficit hyperactivity disorder (ADHD), anxiety, aggression, post-traumatic stress disorder (PTSD) or depression. Sleeping difficulties occurred in 14 individuals. MRIs were performed in 26 individuals, of which seven showed rather non-specific anomalies. Seizures or possible seizures were reported in nine individuals with an onset between 11 months and 10 years.

**Fig. 2:**
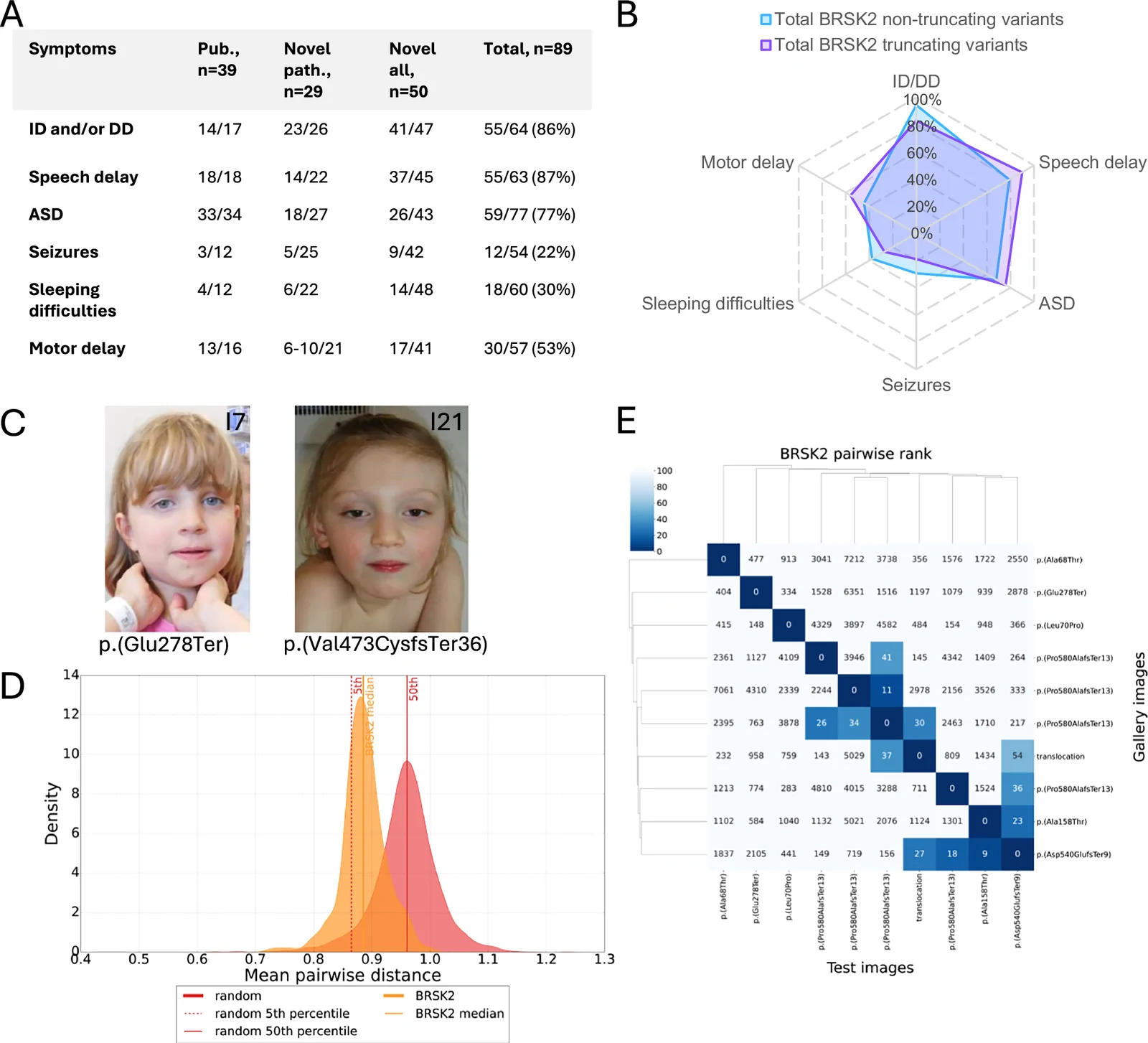
Clinical phenotype. **A** Summary and frequencies of main phenotypes. Pub., published; Path., cases from this cohort with variants classified as pathogenic or likely pathogenic. **B** Phenotype frequencies associated with all novel and published (total *n* = 89) *BRSK2* truncating and missense variants by a radar chart using Canva, indicating no significant differences. **C** Facial photographs of two individuals without major or distinct dysmorphism. **D** *BRSK2* vs. random intra-cohort distance distributions. Kernel density estimates (KDEs) of mean pairwise distances for the *BRSK2* cohort (*n* = 494 cohort-level means) and the random reference (*n* = 3540). Vertical lines mark the random 5th percentile (0.8649; dashed), the random median (0.9603; solid), and the *BRSK2* median (0.8852; solid). Mapping *BRSK2* values to the random ECDF places the *BRSK2* median at the 7.60th percentile of random (cohort percentile summary: median 7.60%, mean 11.83%, range 0.20–82.85%). These results indicate moderate enrichment toward the low-distance tail (greater similarity) relative to random. **E** *BRSK2* pairwise ranks in the GestaltMatcher gallery. Matrix read column-wise: each column is a *BRSK2* query image; each row is a candidate *BRSK2* image. Entries show the returned gallery rank for the row when the column is queried (smaller ranks = stronger similarity). A tight low-rank block is visible for the three related patients carrying p.(Pro580Alafs*13), and additional cross-family low ranks appear sporadically, indicating localized but weaker across-family cohesion. Notably, the p.(Ala158Thr) and p.(Asp540Glufs*9) images reciprocally retrieve each other with rank 9 and rank 23, respectively.

Variable and infrequent other anomalies included muscular hypotonia (11/47), cardiac (3/39) and eye (12/46) abnormalities, microcephaly in four individuals, macrocephaly in three individuals, feeding difficulties, or recurrent infections. Summarized and more detailed clinical information can be found in Fig. 2A and Supplementary Table S1. There was no apparent phenotypic difference between individuals with truncating or missense variants (Fig. 2B).

### Computational facial intra-cohort similarity

Subtle facial aspects were reported commonly, however, without a recognizable distinct gestalt for experienced clinicians. Facial aspects of two individuals are depicted in Fig. 2C. For further analysis we utilized GestaltMatcher [25, 34] to analyse facial similarity between ten individuals with *BRSK2* variants, including three from the same family (variant p.(Pro580Alafs*13)). Compared with a random reference, the *BRSK2* intra-cohort distance distribution shows a mild leftward shift (Fig. 2D). The random reference had a 5th percentile of 0.8649 and a median of 0.9603, whereas the *BRSK2* cohort showed a mean of 0.8860 and a median of 0.8852. Mapping *BRSK2* distances to the random Empirical Cumulative Distribution Function (ECDF) placed the *BRSK2* median at the 7.60th percentile of random (cohort percentile summary: median 7.60%, mean 11.83%, range 0.20–82.85%). Using the prespecified random 5th-percentile threshold (0.8649) provides context for the distribution, but, unlike very cohesive cohorts, the *BRSK2* median lies above this cut-off, consistent with moderate within-cohort similarity. We also assessed image-level similarity using pairwise ranks in the GestaltMatcher gallery (Fig. 2E). The matrix is read column-wise: each column is a query image and each row a candidate; cells are the gallery ranks returned for that query (lower rank = higher similarity). Consistent low ranks are observed within the family harboring p.(Pro580Alafs*13), as expected for shared familial traits. In addition, several cross-family matches reach low ranks, indicating localized cohesion across different individuals with *BRSK2* variants, albeit weaker than in strongly clustered cohorts. However, individuals carrying variants p.(Ala68Thr), p.(Glu278Ter), and p.(Leu70Pro) look relatively not similar to the others. Together, the distributional and rank-based findings support the presence of a *BRSK2*-related facial gestalt with modest but detectable intra-cohort similarity.

### Altered dosage of *sff* leads to neurological phenotypes in flies

To investigate the role of BRSK2 in the nervous system and to model its loss in vivo, we manipulated its orthologue *sugar free frosting* (*sff*) in *Drosophila melanogaster*. We first confirmed reduced *sff* expression to 20% upon ubiquitous knockdown with RNAi-line 2, to 50% residual expression by using RNAi-line 3 and 70% residual expression by using RNAi-line 1 (Supplementary Fig. S2A). In accordance with weaker knockdown efficiency shown by quantitative RT-PCR (Supplementary Fig. S2A), knockdown with RNAi-lines 1 or 3 did not impair viability (Supplementary Fig. S3A), while ubiquitous knockdown with the strongest RNAi-line 2 (Supplementary Fig. S2A) resulted in almost complete lethality (Supplementary Fig. S3A). To avoid lethality, we worked with tissue-specific dosage manipulation and investigated the effect of tissue-specific knockdown of *sff* in either all neurons (pan-neuronal) or glia cells (glial) on locomotor behavior with the negative geotaxis assay and on seizure susceptibility with the bang sensitivity assay.

**Fig. 3:**
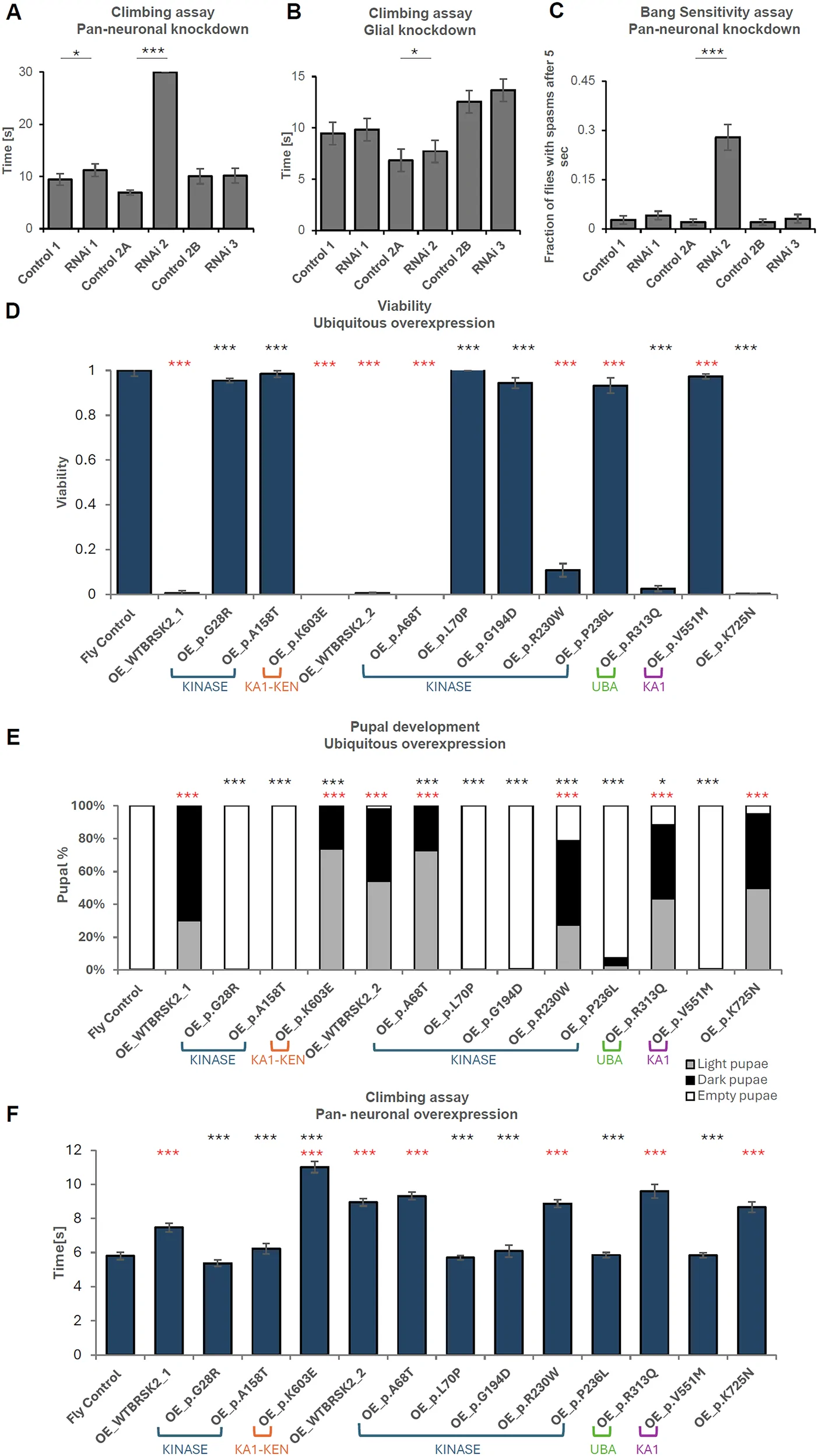
*Sff* or *BRSK2* dosage affects viability and neurological function in flies. **A** Pan-neuronal knockdown with the strongest RNAi-line 2 lead to significantly impaired locomotor behavior in the climbing assay compared to control 2 (number of flies tested per genotype: RNAi n = 200; control *n* = 200). **B** Glial knockdown with RNAi-line 2 lead to mild impairment in locomotor behavior (RNAi *n* = 200; control *n* = 200). **C** Pan-neuronal knockdown with the strongest RNAi-line 2 resulted in significant seizure susceptibility in the bang sensitivity assay (RNAi *n* = 200; control *n* = 200). Control 2 A and Control 2B are the same control line used in two independent experiments. **D** Complete lethality upon ubiquitous overexpression of WT BRSK2, while viability was unimpaired or only partially impaired upon overexpressing mutant BRSK2 with five of seven missense variants from the kinase domain and p.(Val551Met) from the KA1-domain. A minimum of 200 flies per genotype was tested. Graphs show mean values for control and overexpression lines with standard deviation. Statistical testing was done with two tailed t-test. **E** Light (early developmental stop), dark (late developmental stop) and empty (hatched), non-TB pupae were counted. Significant fewer light pupae for p.(Arg230Trp) from the kinase domain and p.(Arg313Gln) from the UBA domain, compared to the WT, indicate a partial loss-of-function effect. Significantly more light pupae for the KA1-domain-KEN-Box domain variant p.(Lys603Glu) indicate a possible gain-of-function effect. A minimum of 200 pupae per genotype was counted. Statistical testing was done with two tailed student’s t-test **D, E**. **F** Locomotor impairment upon pan-neuronal overexpression of WT *BRSK2*, while overexpression of mutant BRSK2 did not siginificantly impair locomotor behavior for six variants. Locomotor impairment upon overexpressing *BRSK2* with the KA1-domain variant p.(Lys603Glu) is more severe than in WT. A minimum of 200 flies was tested per genotype. Graphs show mean values for control, RNAi and overexpression lines with standard error mean (SEM), and significance was tested using Wilcoxon signed-rank test **A–C, F**. Asterisks indicate statistical significance (∗*p *< 0.05 ∗∗*p* < 0.01, ∗∗∗*p* < 0.001). In Figure panels **D–F**, red asterisks indicate comparison to the control line, and black asterisks indicate comparison to the overexpression of the respective Wildtype (OE_WTBRSK2).

Tissue-specific pan-neuronal or glial knockdown with the strongest RNAi-line 2 resulted in impaired locomotor behavior, which was more pronounced upon pan-neuronal than glial knockdown (Fig. 3A, B). Pan-neuronal knockdown with RNAi-line 2 also resulted in significant seizure susceptibility (Fig. 3C), while glial knockdown did not result in a bang sensitivity phenotype (Supplementary Fig. S3B).

As only knockdown below 20% residual levels with RNAi-line 2 resulted in lethality and neurological phenotypes, while knockdown with two weaker lines did not result in significant phenotypes, this indicates a tightly regulated *sff* dosage. The observed phenotypes being an off-target effect of RNAi-line 2 is rather unlikely, as similar phenotypes were described in an *sff* mutant line [35].

### Most missense variants in the kinase domain result in loss of function

As loss of function or haploinsufficiency is more predictable for truncating variants or deletions, consequences of missense variants are often less obvious, thus challenging their interpretation. We therefore utilized *Drosophila* to investigate eleven missense variants from this study. First, we overexpressed human *BRSK2* in a *sff* deficient fly background to test if the lethality and gross neurological phenotypes observed upon ubiquitous or tissue-specific *sff* knockdown can be rescued by overexpression of wildtype or mutant B*RSK2* constructs. However, both the toxic effect of overexpressing wildtype BRSK2 and the lethality upon *sff* knockdown prevented further testing (Supplementary Fig. S4).

We then overexpressed wildtype or mutant *BRSK2* in a wildtype fly background. First, transgene expression was confirmed for all overexpression lines, with the majority of mutant constructs showing RQ Mean values within a comparable range to OE_WTBRSK2 lines (Supplementary Fig. S2B). Variable transgene expression of single lines influencing the subsequent assays’ results cannot be ruled out. Overexpression of wildtype *BRSK2* resulted in partial lethality in prepupal and pupal (Supplementary Fig. S5) stages and complete lethality in adult stages (Fig. 3D), thus indicating a toxic effect. Overexpression of *BRSK2* containing any of the five missense variants p.(Gly28Arg), p.(Leu70Pro), p.(Ala158Thr), p.(Gly194Asp), p.(Pro236Leu) from the kinase domain or one variant p.(Val551Met) from the KA1 domain did not impair viability, therefore being compatible with a loss-of-function effect (Fig. 3D–E). In addition to a trend of more surviving adult flies, assessment of lethality in pupal stages revealed a significant lower number of light pupae (early pupal death) for variants p.(Arg230Trp) from the kinase domain and p.(Arg313Gln) from the UBA domain. This might point to partial loss of function for these two variants (Fig. 3E).

In contrast, overexpression of *BRSK2* containing missense variants (p.Ala68Thr) from the kinase domain, p.(Lys603Glu) from the KA1 domain (KEN box), or the C-terminal variant p.(Lys725Asn) resulted in a similar lethality as the wildtype indicating absence of a loss-of-function effect (Fig. 3D). However, when we assessed viability/lethality at pupal stages, we observed a significantly larger fraction of light pupae or non-Tb pupae upon overexpression of BRSK2 containing any of these variants compared to wildtype BRSK2, indicating an increased toxicity. This would be compatible with a gain-of-function effect (Fig. 3E and Supplementary Fig. S5).

When overexpressing BRSK2 pan-neuronally and assessing locomotor behavior of viable flies in the negative geotaxis assay, we observed similar loss-of-function effects for the five missense variants from the kinase domain and one KA1 domain variant. Of note, the second missense variant from the KA1 domain, p.(Lys603Glu), was associated with a more severe locomotor impairment than the wildtype, which would be support a gain of function in terms of increased toxicity (Fig. 3F). The four remaining missense variants did not result in a significantly altered locomotor behavior (Fig. 3F) (Supplementary Table S2).

## Discussion

By assembling 52 cases (50 of them previously unreported), we further delineate the molecular and clinical spectrum of *BRSK2*-associated NDD. The clinical phenotype is rather non-specific and variable with borderline to moderate developmental delay/ID, ASD or other behavioral issues. Of note, the frequency of autism or ASD in this study is lower than in previous reports (26/43 versus 33/34). This might be due to an ascertainment bias as most of the previous reports were from large ASD cohorts. Various and variable anomalies such as seizures, hypotonia, or visual impairment can occur, but do not result in a distinct syndromic pattern. Subtle facial features were frequently observed among affected individuals; however, these characteristics did not coalesce into a consistent or recognizable facial gestalt. However, facial analysis on ten individuals with available frontal photos using GestaltMatcher identified several similarities among unrelated individuals, which might contribute to variant interpretation and confirmation of diagnosis in the future. As some individuals, e.g. family members of index cases, were reported with normal intelligence, penetrance for neurodevelopmental deficits and cognitive impairment appears to be incomplete. This and the generally rather mild disease expression is in accordance with a relatively large proportion of inherited variants in our cohort and with observing some of the variants in low frequencies in the gnomAD database. It also has to be considered that some of the observed variants might actually not be disease relevant.

Of note, additional variants in other disease-associated genes were observed in 15 individuals. One was a recurrent 16p11.2 deletion, ten variants were in genes also associated with NDDs and classified as VUS, and several were de novo variants in not (yet) disease associated genes. These variants, in combination with the *BRSK2*-variant, might contribute to the NDD phenotype in some cases.

Both BRSK1 and BRSK2 play a crucial role in neuronal polarization, migration and axonogenesis, highly important neuronal processes [4]. Recently, also haploinsufficiency of *BRSK1* has been discovered to be associated with epilepsy and NDD [36]. *BRSK2* knockout in mice resulted in early postnatal lethality and disturbances in cortical development [29]. In *brsk2b*-mutant zebrafish, impairment in locomotor and social behavior were observed [11]. *Sff*-mutant *Drosophila* showed impairment in locomotor behavior [35]. Similarly, ubiquitous or tissue-specific knockdown of *sff* to a residual level of 20% resulted in lethality or neurological phenotypes, respectively, confirming its role in development and neurological function as well as its dosage sensitivity, matching to the high pTriplo and pHaplo scores for its orthologue *BRSK2* [37]. *BRSK2* expression is higher in neuronal cells than in glial cells [38], which is in accordance with more severe fly phenotypes upon pan-neuronal than upon glial knockdown. In line with epilepsy occurring in about 20% of individuals with *BRSK2* variants, we also observed seizure susceptibility in flies with pan-neuronal knockdown of *sff*. Pan-neuronal knockdown of *sff* therefore appeared to model the human NDD due to haploinsufficiency quite well, without, however, providing insights into specifically affected pathways or processes.

Additionally, we utilized the fly to investigate the effects of the missense variants collected in our cohort. Conditions with incomplete penetrance and high variability usually challenge the interpretation of identified variants. Loss-of-function effects or haploinsufficiency can be assumed for the majority of truncating variants [39]. However, pathogenicity of missense variants often remains unclear, particularly in haploinsufficiency intolerant genes, and they less often cluster in functional domains compared to a gain-of-function or dominant-negative context [40]. Next to parameters such as frequency, segregation and in-silico predictions, increasing importance comes to further analyses such as structural modeling of variant effects and functional assays [41]. *Drosophila* is an established model to characterize and validate disease related candidate variants from humans and to investigate possible loss-of-function, gain-of-function or dominant negative effects, as demonstrated in numerous studies [42–45]. One approach is to overexpress the human wildtype or mutant gene in a normal fly background and to assess degrees of toxicity, the other approach is to overexpress the human wildtype or mutant gene in a fly deficient background and to assess rescue capabilities. While the second approach was not applicable in our study, we could still test the first approach. We found evidence for loss of function for five of the seven kinase domain missense variants and a KA1 domain variant as there were no or only mild effects on lethality and neurological behavior compared to the toxicity observed for the wildtype when they were overexpressed. For p.(Lys603Glu) from the KA1-KEN Box domain, fly experiments indicated a gain-of-function effect in two assays. Therefore, we applied the PS3_moderate criterion on these variants, which, however, contributed to classification as likely pathogenic only for one variant and still resulted in the classification as VUS for the other five. The remaining variants showed mild loss-of-function or gain-of-function effects in only one of the experimental settings. This supports their disease-causing relevance, but is, in our opinion, not definitive enough to apply any strength of PS3 and therefore contribute to classification.

Our observations demonstrate that prediction of the effect of missense variants with in silico tools or structural modeling might differ from functional analyses. While haploinsufficiency from loss of function due to protein truncating or impairment of the kinase domain by missense variants is the most likely mechanism for the majority of variants in *BRSK2* and also predicted by structural modeling for all missense variants, we found functional evidence that not all missense variants in BRSK2 might have the same effect, and that also gain of function might contribute to the pathomechanism. No obvious phenotypic differences could be noted regarding variants with different functional consequences. Despite utilizing a variety of in silico and in vivo functional tools, classification of missense variants remains still challenging.

A comprehensive variant interpretation requires a broad spectrum of approaches, and functional testing might need to be tailored to each variant’s molecular context. It also has to be considered that in vivo functional results from a *Drosophila* approach like this might contribute to variant validation but do not provide mechanistic insights into the variant’s specific consequences in terms of loss of kinase activity, dominant-negative interference or gain of function at a biochemical level, neither into effects on downstream pathways such as mTORC. These questions have to be addressed in future studies with more cell-based experimental approaches.

## Conclusion

In summary, our findings further delineate the clinical and molecular spectrum of *BRSK2*-associated NDD. They further characterize the role of BRSK2/sff in nervous system function and the pathogenic relevance of missense variants depending on their location.

## Supplementary information

Supplementary material

Supplemetary Table S1

Supplementary Table S2

Supplementary Table S5

## Data Availability

The datasets included and analysed during this study are available from the corresponding author upon reasonable request. Identified variants in *BRSK2* were submitted to ClinVar.
