# Supplementary material for: Further characterization of the BRSK2-associated neurodevelopmental disorder
Source: Eur J Hum Genet. 2026 Jul 27;34(11):1644–53. doi: 10.1038/s41431-026-02195-7 (PMC13743746; doi:10.1038/s41431-026-02195-7)
Supplement: Supplementary file 1 [file 41431_2026_2195_MOESM1_ESM.pdf]

SUPPLEMENTARY DATA

SUPPLEMENTARY METHODS

Facial gestalt and similarity analysis

We evaluated facial similarity using the GestaltMatcher framework (1, 2). Publication-consented photographs were processed to compute ensemble Facial Phenotype Descriptors (FPDs), and pairwise distances (smaller = more similar) were derived in the Clinical Face Phenotype Space. To construct the *BRSK2* intra-cohort distribution, we sampled one image per family and computed the mean pairwise distance for each such cohort configuration, yielding 494 cohort-level means (n_*BRSK2* = 494). As a null reference, we generated a random distribution by repeatedly sampling matched-size cohorts from a gallery of 1,499 images spanning 321 disorders from GestaltMatcher Database (GMDB) (3) and computing the cohort mean for each draw (n_random = 3540). Kernel density estimates were plotted for *BRSK2* and the random reference, with vertical lines marking the random 5th percentile (0.8649), the random median (0.9603), and the *BRSK2* median (0.8852). Each *BRSK2* mean distance was mapped to its right-inclusive percentile in the random ECDF; we report the median, mean, and range of these percentiles. For image-level validation, we performed a pairwise rank analysis in which every *BRSK2* image served as a query against the GMDB gallery. For each query, we recorded the gallery rank of every other *BRSK2* image. The resulting column-wise rank matrix highlights within-cohort retrieval performance and visually localizes familial versus cross-family cohesion.

Behavioural experiments

Seizure susceptibility was tested with the bang sensitivity assay. Paralytic behaviour and hyperactivity can be induced by a mechanical shock such as vortexing for 10 s (4). *Sff* RNAi lines or *BRSK2* overexpression lines or *BRSK2* OE/*sff* RNAi double balancer lines and their corresponding genetic background control lines were crossed to the *elav*-GAL4/Cyo (pan-neuronal) or to the *repo*-GAL4/Tm3Sb (glial) driver lines. From the offspring, a minimum of 200 flies per genotype and corresponding controls were collected 0–48 hrs after eclosion

under CO_2 anesthesia in groups of ten (with a balanced male/female ratio) and kept in normal food vials for 24 hrs. The next day, pseudonymized groups of flies were flipped into a vial without food, were allowed to recover for 1 min and were vortexed for 10 s at maximum strength. The number of flies displaying paralysis or spasms 5 s after vortexing was assessed. The negative geotaxis assay/climbing assay is a quantitative test to analyse the locomotor function of the fly (5). Crosses and collection of flies were done as described for the bang-sensitivity assay. After transfer to testing vials with an adjustment time of 1 min, flies were tapped to the bottom of the vial and videotaped for 30 s. Time was measured until 70% of flies had climbed 8.8 cm towards a light source. Data from bang sensitivity and climbing assay were averaged respectively, and testing for statistical significance was performed with a two-tailed Wilcoxon signed-rank test in R version 4.2.0.

Databases, Resources and Tools

Genematcher (6)

VarSome (7)

DECIPHER (8)

ACMG criteria (9)

ClinVar (10)

Clustalw2 (11, 12)

Prosite (13)

ELM database (14)

AlphaMissense (15)

Bloomington Drosophila Stock Center (16)

Viennea Drosophila Research Center (17)

FlyORF (Zurich, Switzerland) (18)

DIOPT (19, 20)

SUPPLEMENTARY FIGURES

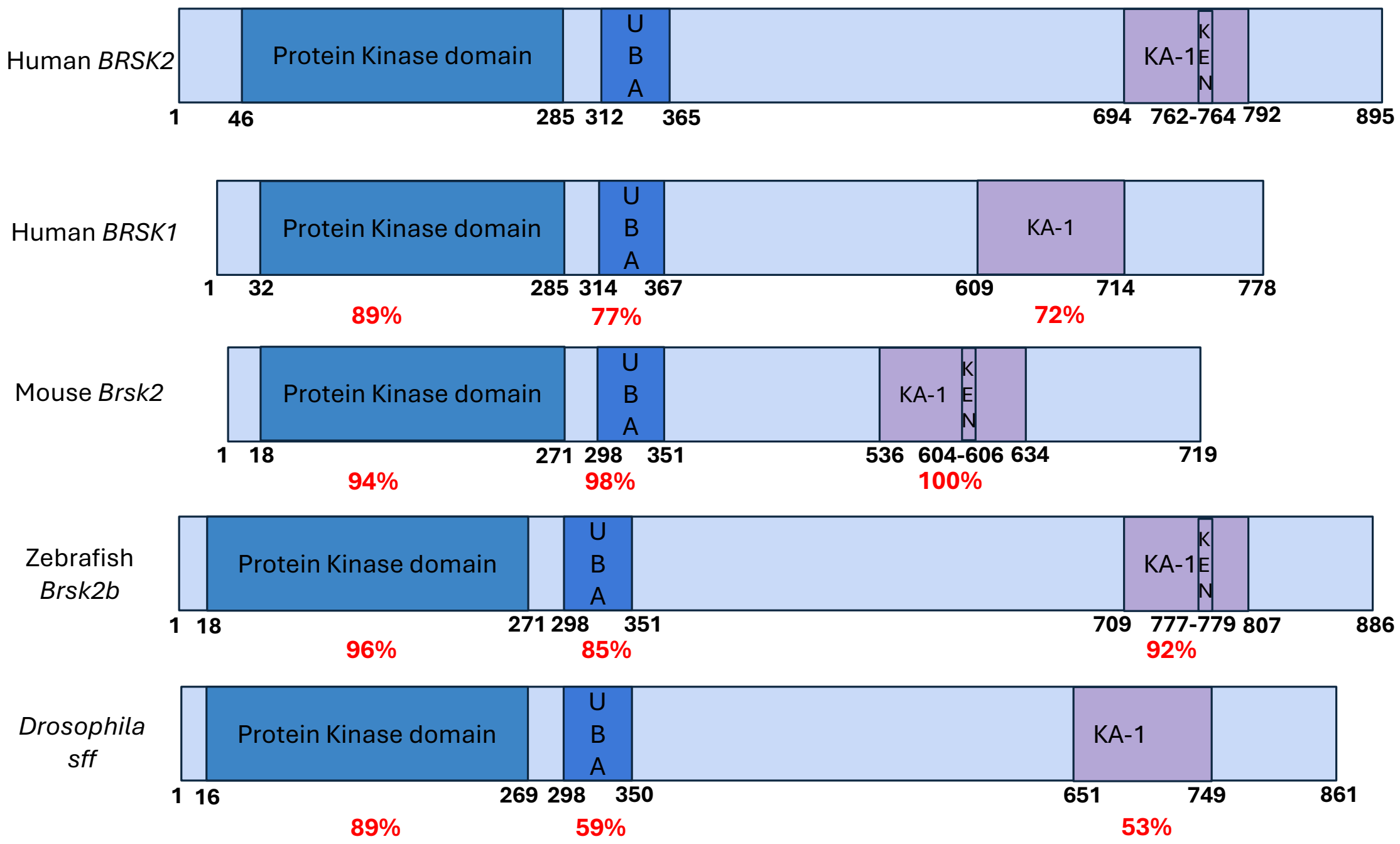

Figure S1 Schematic overview of human BRSK2, BRSK1 and its orthologues across species, showing conservation of functional domains.

Conservation analyses were performed using DIOPT, with the following reference sequences: human BRSK2 (XP_054189097.1), human BRSK1 (NP_115806.1), mouse Brsk2 (NP_001009930.1), zebrafish Brsk2b (XP_009301700.1) and *Drosophila* sff (NP_648814.3). Each bar represents the full-length protein of the indicated species. All orthologues share three conserved domains: an N-terminal Protein Kinase domain, a UBA (Ubiquitin-Associated) domain, and a C-terminal KA-1 (Kinase Associated-1) domain containing KEN BOX. Red percentages indicate amino acid identity to human BRSK2 for each domain region, demonstrating highest conservation in the Protein Kinase domain (89-96%) and with variable conservation in UBA domain (59-98%), and in the KA-1 domain (53-100%).

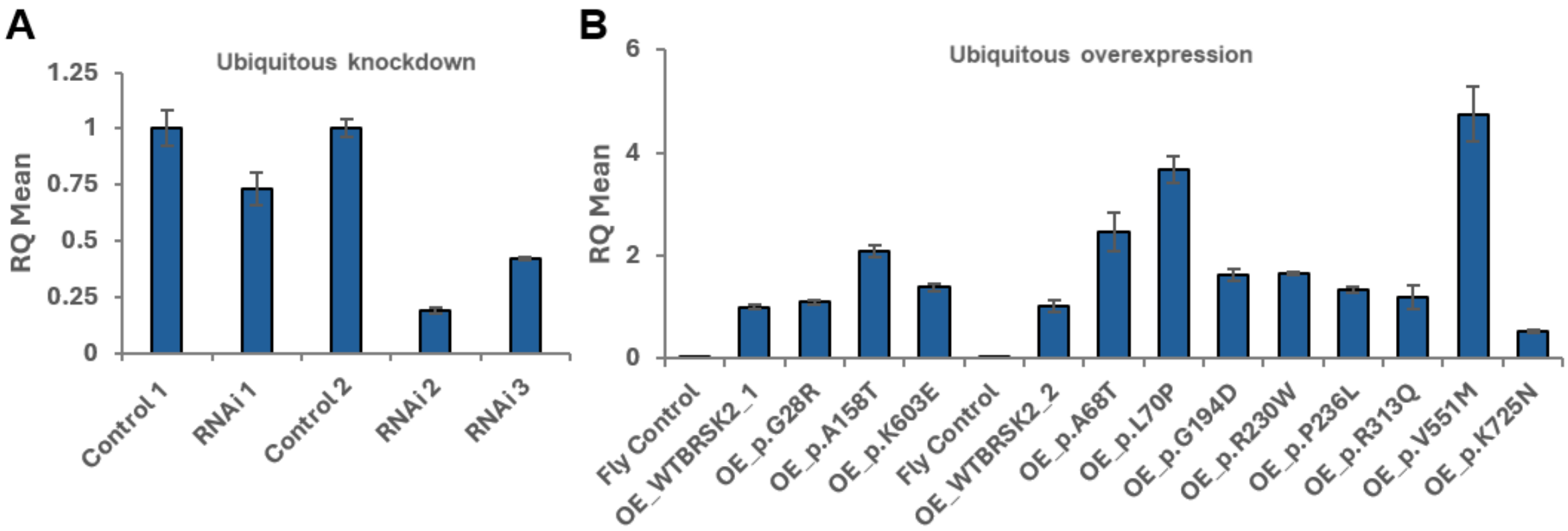

Figure S2 Confirmation of *sff* knockdown and *BRSK2* overexpression in flies by quantitative RT-PCR.

A RNAi-mediated, ubiquitous (*Actin*-GAL4) knockdown of *sff* with RNAi-line 1 resulted in 70% residual expression levels compared to control 1. Knockdown with RNAi-line 2 and RNAi-line 3 resulted in 20% and 50% residual levels, respectively, compared to the corresponding control 2. **B** Ubiquitous overexpression of WT or mutant *BRSK2* constructs, the latter carrying one of eleven missense variants and compared to Fly Control. Relative expression (RQ) values were calculated using the ΔΔCt method with corresponding controls as reference. Bars represent RQ values, error bars represent the standard deviation of the RQ value.

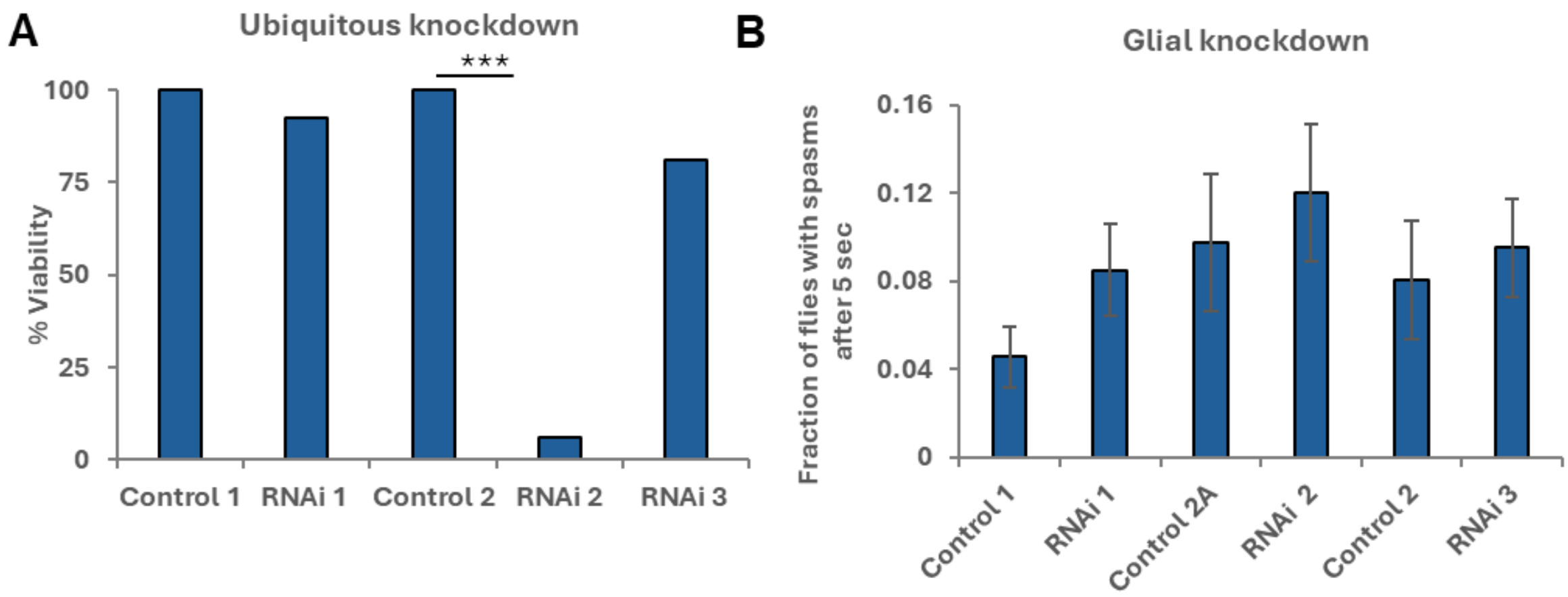

Figure S3 Viability and Bang sensitivity of flies with ubiquitous or glial dosage manipulation of *sff.*

A Ubiquitous knockdown with RNAi-line 2 (strongest knockdown) leads to nearly complete lethality, while knockdown with RNAi-lines 1 and 3, respectively, does not significantly impair viability. Statistical testing was done with chi-square test, comparing Sb (Balancer) vs. non-Sb (knockdown) flies (*** $p < 0.001$). **B** Glial knockdown with RNAi lines 1-3 did not lead to seizure susceptibility. Graphs show mean values for control and RNAi lines with standard error mean and significance was tested using Wilcoxon signed-rank test. Control 2A and Control 2B are the same control line used in two independent experiments.

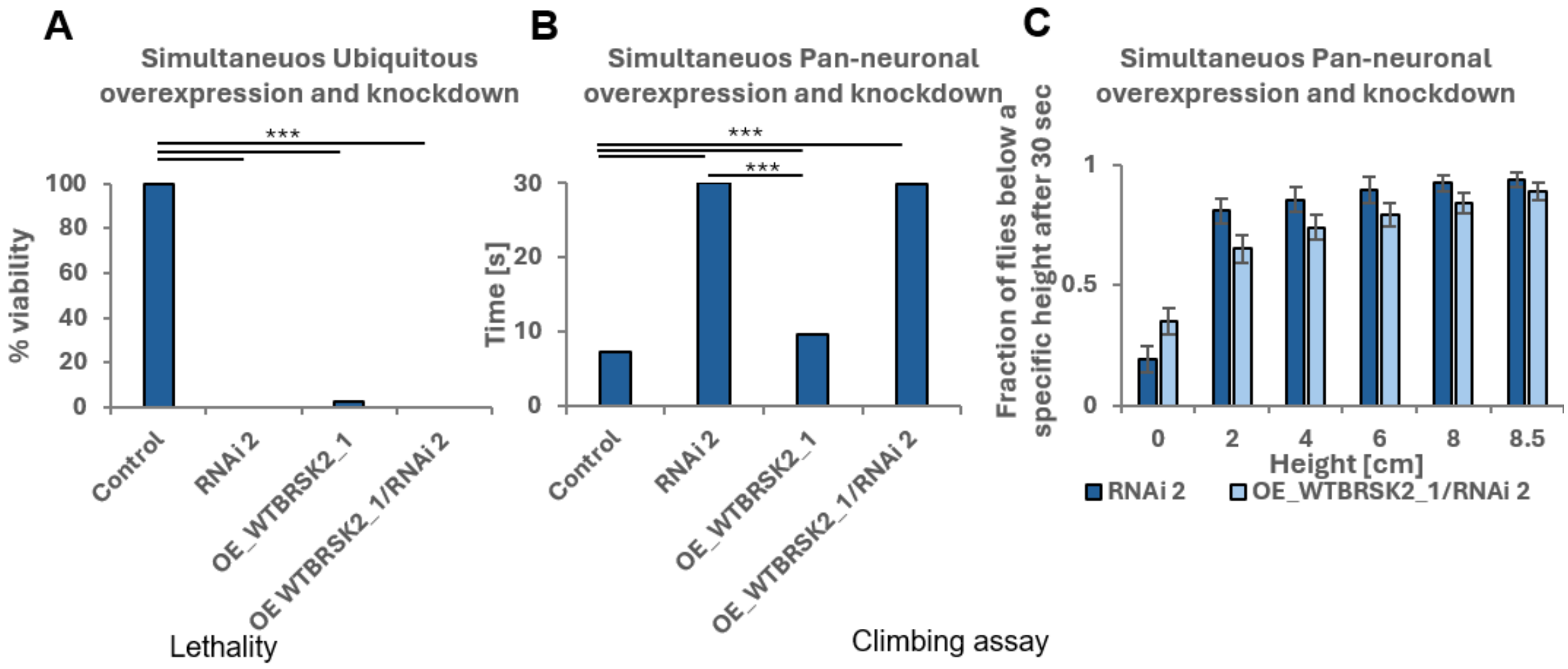

Figure S4 *BRSK2* overexpression cannot rescue *sff* deficiency due to toxicity.

A Lethality resulting from ubiquitous *sff* knockdown with RNAi-line 2 cannot be rescued upon simultaneous overexpression of WT BRSK2. At least 150 adult flies were counted for each line. Statistical analysis was done using Chi-square test (*** $p < 0.001$). **B** The locomotor phenotype was not rescued upon simultaneous pan-neuronal knockdown with sff RNAi-line 2 and overexpression with WT_BRSK2_1. Either due to the severe phenotype upon pan-neuronal *sff* knockdown or mild phenotype upon overexpression of WT BRSK2 this phenotype cannot be rescued upon simultaneous overexpression of WT BRSK2. Graphs show mean values for control and overexpression lines with standard error mean and significance was tested using Wilcoxon signed-rank test. **C.** Due to severity of the locomotor phenotype upon *sff* knockdown, another assessment was done where fraction of flies which were unable to climb certain heights (0cm, 2cm, 4cm, 6cm, 8cm) of the vial after 30 sec were counted.

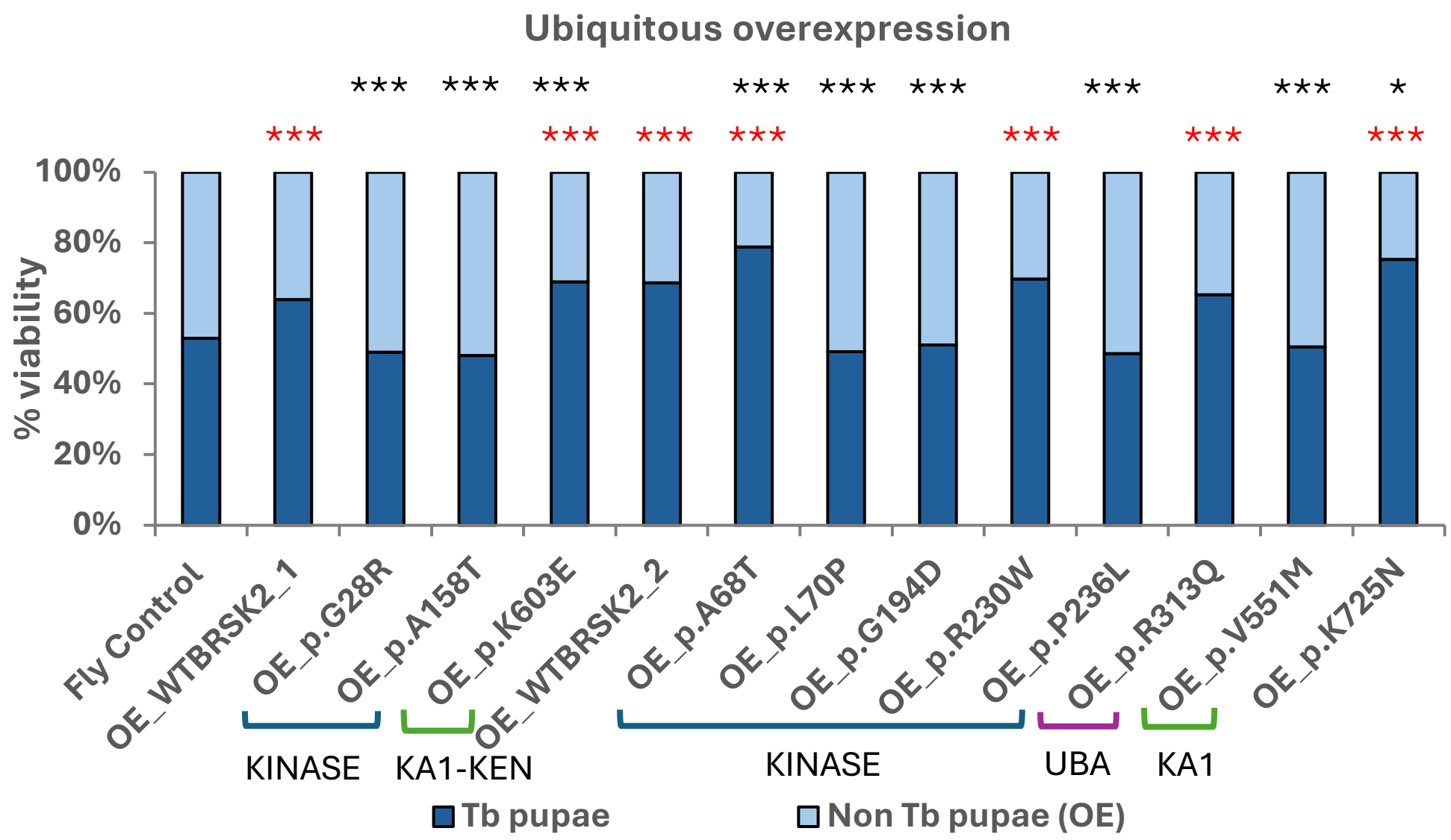

Figure S5 Pupal viability upon overexpression of wildtype or mutant *BRSK2* in flies. Viability upon ubiquitous (Actin-GAL4/Tm3 Sb Tb) overexpression of *BRSK2* WT or mutant constructs was assessed at the pupal stages. Under normal conditions, the expected ratio of balancer (Tb) versus non-balancer (Non Tb) progeny is 1:1 (50%). At least 150 pupae were counted for each line. At the pupal stage, overexpression of WT *BRSK2* led to partial pre-pupal lethality, with approximately 65-70% of pupae carrying the balancer chromosome. Overexpression of *BRSK2* constructs harbouring five of the seven missense variants within the kinase domain and variant p.(Val551Met) from the KA1 domain did not impair pupal development. Overexpression of BRSK2 with variant p.(Arg230Trp) from the kinase domain, p.(Arg313Gln) from the UBA domain and p.(Lys725Asn) from the c-terminus led to partial pre-pupal lethality similar to WT. Overexpression of the BRSK2 construct containing (p.Ala68Thr) from the kinase domain, the KA1-KEN Box variant p.(Lys603Glu) and the C-terminal variant p.(Lys725Asn) caused significantly more partial pre-pupal lethality in comparison to the WT. Statistical testing was done with chi-square test (*** $p < 0.001$, * $p < 0.05$). Red asterisks indicate comparison to the control line, and black asterisks indicate comparison to the overexpression of the respective wildtype (OE_WTBRSK2).

SUPPLEMENTARY TABLES

Table S1 Clinical and Molecular data

(excel sheet)

Table S2 Pathogenicity Predictions

(excel sheet)

Splice prediction: SpliceAI (21) and Pangolin (22)

Missense variant prediction: REVEL (23), SIFT (max) (24), PrimateAI-3D (25), CADD (26) and phyloP (27), AlphaMissense (15).

Table S3. Overview of fly lines used in the study

Fly lines	description	identifier	source	corresponding control line
**Control lines**				
w1118 next generation	Control 1	60100	VDRC	
y[1] v[1]; P{y[+t7.7]=CaryP}attP2	Control 2	BL#36303	BDSC	
y1 M{vas-int.Dm}ZH-2A w*; M{3xP3-RFP.attP}ZH-86Fb	Fly Control	BL#24749	BDSC	
***sff* RNAi lines**				
P{KK108085}VIE-260B	RNAi-line 1	100717/KK	VDRC	60100
y1 sc* v1 sev21; P{TRiP.HMS01280}attP2	RNAi-line 2	BL#36656	BDSC	BL#36303
y1 v1; P{TRiP.HMC04176}attP2	RNAi-line 3	BL#55897	BDSC	BL#36303
**Driver lines**				
Actin-Gal4/ Tm3Sbtb	ubiquitous driver, chr. 3		colleagues	
Elav-Gal4/ CyO	pan-neuronal driver, chr. 2	BL#8765	BDSC	
Repo-Gal4/ Tm3 sb	Glial driver, chr. 2	BL#7415	BDSC	
**Double balancer lines**				
Kr/Cyo;D/Tm6c sb tb			colleagues	
**BRSK2 fly lines**				
OE BRSK2-WT1	OE_WTBRSK2_1		FlyORF	BL#24749
OE BRSK2-MG82	OE_p.G28R		FlyORF	BL#24749
OE BRSK2-MG472	OE_p.A158T		FlyORF	BL#24749
OE BRSK2-MG1807	OE_p.K603E		FlyORF	BL#24749
OE BRSK2-WT2	OE_WTBRSK2_2		FlyORF	BL#24749
OE BRSK2-MG202	OE_p.A68T		FlyORF	BL#24749
OE BRSK2-MG209	OE_p.L70P		FlyORF	BL#24749
OE BRSK2-MG581	OE_p.G194D		FlyORF	BL#24749
OE BRSK2- MG688	OE_p.R230W		FlyORF	BL#24749
OE BRSK2- MG707	OE_p.P236L		FlyORF	BL#24749
OE BRSK2-MG938	OE_p.R313Q		FlyORF	BL#24749
OE BRSK2- MG1651	OE_p.V551M		FlyORF	BL#24749
OE BRSK2-MG2175	OE_p.K725N		FlyORF	BL#24749
**Double balancer constructs**				
BRSK2 WT; 36656			This study	
BRSK2 MG82; 36656			This study	
BRSK2 MG472; 36656			This study	

BRSK2 MG1807 / (CyO); 36656 / (Tm6c)			This study	

Table S4 List of Primers used in the study

Primer	Sequence	Remarks
BRSK2_qF1	GGCCATCAAGATCGTCAACC	qPCR/RT-PCR *Drosophila*
BRSK2_qR1	CACCTGACACGTGTTCTAGC	qPCR/RT-PCR *Drosophila*
BRSK2_qF2	GCGGCTCAACTCCATCAAG	qPCR/RT-PCR *Drosophila*
BRSK2_qR2	ACCACGAAGATCTGCTCCTC	qPCR/RT-PCR *Drosophila*
BRSK2_MG_82_F	GACGCTGGGCAAGCGGCAGACAGGTC	mutagenesis
BRSK2_MG_82_R	GACCTGTCTGCCGCTTGCCCAGCGTC	mutagenesis
BRSK2_MG_202_F	GAGCGGGAGATCACGATCCTGAAGC	mutagenesis
BRSK2_MG_202_R	GCTTCAGGATCGTGATCTCCCGCTC	mutagenesis
BRSK2_MG_209_F	GAGATCGCGATCCCGAAGCTCATTGAG	mutagenesis
BRSK2_MG_209_R	CTCAATGAGCTTCGGGATCGCGATCTC	mutagenesis
BRSK2_MG_472_F	GAACAACATCCGCATCACAGACTTTGGCATGG	mutagenesis
BRSK2_MG_472_R	CCATGCCAAAGTCTGTGATGCGGATGTTGTTC	mutagenesis
BRSK2_MG_581_F	GAGAAGTATGACGACCGGAAGGCGGAC	mutagenesis
BRSK2_MG_581_R	GTCCGCCTTCCGGTCGTCATACTTCTC	mutagenesis
BRSK2_MG__688_F	GAGAAGGTGAAGTGGGGCGTGTTC	mutagenesis
BRSK2_MG__688_R	GGAACACGCCCCACTTCACCTTCTC	mutagenesis
BRSK2_MG__707_F	CGTGTTCCACATGCTGCACTTTATCCCG	mutagenesis
BRSK2_MG__707_R	CGGGATAAAGTGCAGCATGTGGAACACG	mutagenesis
BRSK2_MG__938_F	CTGGGCTGCTTCCAAGACCGCAACAAG	mutagenesis
BRSK2_MG__938_R	CTTGTTGCGGTCTTGGAAGCAGCCCAG	mutagenesis
BRSK2_MG__1651_F	CAAGGCTGACATCATGCACGCCTTCC	mutagenesis
BRSK2_MG__1651_R	GGAAGGCGTGCATGATGTCAGCCTTG	mutagenesis
BRSK2_MG_1807_F	GTGGGGAGGCGCAGGAGGAGAACGGCATC	mutagenesis
BRSK2_MG_1807_R	GATGCCGTTCTCCTCCTGCGCCTCCCCAC	mutagenesis
BRSK2_MG__2175_F	GGACACGGCCAACATGGGCCCGCCC	mutagenesis
BRSK2_MG__2175_R	GGGCGGGCCCATGTTGGCCGTGTCC	mutagenesis

Supplementary Table S5 Additional variants in disease associated genes

(excel sheet)

REFERENCES

1. Hsieh TC, Bar-Haim A, Moosa S, Ehmke N, Gripp KW, Pantel JT, et al. GestaltMatcher facilitates rare disease matching using facial phenotype descriptors. Nature Genetics. 2022;54(3):349-+.
2. Hustinx A, Hellmann F, Sümer Ö, Javanmardi B, André E, Krawitz P, et al. Improving Deep Facial Phenotyping for Ultra-rare Disorder Verification Using Model Ensembles. Ieee Wint Conf Appl. 2023:5007-17.
3. Lesmann H, Hustinx A, Moosa S, Klinkhammer H, Marchi E, Caro P, et al. GestaltMatcher Database - A global reference for facial phenotypic variability in rare human diseases. Res Sq. 2024.
4. Kuebler D, Tanouye MA. Modifications of seizure susceptibility in. J Neurophysiol. 2000;83(2):998-1009.
5. Palladino MJ, Hadley TJ, Ganetzky B. Temperature-sensitive paralytic mutants are enriched for those causing neurodegeneration in drosophila. Genetics. 2002;161(3):1197-208.
6. Sobreira N, Schiettecatte F, Valle D, Hamosh A. GeneMatcher: a matching tool for connecting investigators with an interest in the same gene. Hum Mutat. 2015;36(10):928-30.
7. Kopanos C, Tsiolkas V, Kouris A, Chapple CE, Albarca Aguilera M, Meyer R, et al. VarSome: the human genomic variant search engine. Bioinformatics. 2019;35(11):1978-80.
8. Foreman J, Perrett D, Mazaika E, Hunt SE, Ware JS, Firth HV. DECIPHER: Improving Genetic Diagnosis Through Dynamic Integration of Genomic and Clinical Data. Annu Rev Genomics Hum Genet. 2023;24:151-76.
9. Richards S, Aziz N, Bale S, Bick D, Das S, Gastier-Foster J, et al. Standards and guidelines for the interpretation of sequence variants: a joint consensus recommendation of the American College of Medical Genetics and Genomics and the Association for Molecular Pathology. Genet Med. 2015;17(5):405-24.
10. Landrum MJ, Lee JM, Benson M, Brown GR, Chao C, Chitipiralla S, et al. ClinVar: improving access to variant interpretations and supporting evidence. Nucleic Acids Res. 2018;46(D1):D1062-D7.
11. Goujon M, McWilliam H, Li WZ, Valentin F, Squizzato S, Paern J, et al. A new bioinformatics analysis tools framework at EMBL-EBI. Nucleic Acids Research. 2010;38:W695-W9.
12. Sievers F, Wilm A, Dineen D, Gibson TJ, Karplus K, Li WZ, et al. Fast, scalable generation of high-quality protein multiple sequence alignments using Clustal Omega. Mol Syst Biol. 2011;7.
13. de Castro E, Sigrist CJA, Gattiker A, Bulliard V, Langendijk-Genevaux PS, Gasteiger E, et al. ScanProsite: detection of PROSITE signature matches and ProRule-associated functional and structural residues in proteins. Nucleic Acids Research. 2006;34:W362-W5.
14. Kumar M, Michael S, Alvarado-Valverde J, Zeke A, Lazar T, Glavina J, et al. ELM-the Eukaryotic Linear Motif resource-2024 update. Nucleic Acids Research. 2023;52(D1):D442-D55.
15. Cheng J, Novati G, Pan J, Bycroft C, Zemgulyte A, Applebaum T, et al. Accurate proteome-wide missense variant effect prediction with AlphaMissense. Science. 2023;381(6664):eadg7492.
16. Cook KR, Parks AL, Jacobus LM, Kaufman TC, Matthews KA. New research resources at the Bloomington Drosophila Stock Center. Fly. 2010;4(1):88-91.
17. Dietzl G, Chen D, Schnorrer F, Su KC, Barinova Y, Fellner M, et al. A genome-wide transgenic RNAi library for conditional gene inactivation in. Nature. 2007;448(7150):151-U1.
18. Bischof J, Björklund M, Furger E, Schertel C, Taipale J, Basler K. A versatile platform for creating a comprehensive UAS-ORFeome library in. Development. 2013;140(11):2434-42.
19. Hu Y, Comjean A, Rodiger J, Liu Y, Gao Y, Chung V, et al. FlyRNAi.org-the database of the Drosophila RNAi screening center and transgenic RNAi project: 2021 update. Nucleic Acids Res. 2021;49(D1):D908-D15.

20. Hu Y, Flockhart I, Vinayagam A, Bergwitz C, Berger B, Perrimon N, et al. An integrative approach to ortholog prediction for disease-focused and other functional studies. BMC Bioinformatics. 2011;12:357.
21. Jaganathan K, Kyriazopoulou Panagiotopoulou S, McRae JF, Darbandi SF, Knowles D, Li YI, et al. Predicting Splicing from Primary Sequence with Deep Learning. Cell. 2019;176(3):535-48 e24.
22. Zeng T, Li Y. Predicting RNA splicing from DNA sequence using Pangolin. Genome Biol. 2022;23(1).
23. Ioannidis NM, Rothstein JH, Pejaver V, Middha S, McDonnell SK, Baheti S, et al. REVEL: An Ensemble Method for Predicting the Pathogenicity of Rare Missense Variants. American Journal of Human Genetics. 2016;99(4):877-85.
24. Ng PC, Henikoff S. SIFT: predicting amino acid changes that affect protein function. Nucleic Acids Research. 2003;31(13):3812-4.
25. Sundaram L, Gao H, Padigepati SR, McRae JF, Li YJ, Kosmicki JA, et al. Predicting the clinical impact of human mutation with deep neural networks (vol 50, pg 1161, 2018). Nature Genetics. 2019;51(2):364-.
26. Rentzsch P, Witten D, Cooper GM, Shendure J, Kircher M. CADD: predicting the deleteriousness of variants throughout the human genome. Nucleic Acids Research. 2019;47(D1):D886-D94.
27. Pollard KS, Hubisz MJ, Rosenbloom KR, Siepel A. Detection of nonneutral substitution rates on mammalian phylogenies. Genome Res. 2010;20(1):110-21.
